# Developing Client Self-Advocacy in Occupational Therapy: Are We Practicing What We Preach?

**DOI:** 10.1155/2024/1662671

**Published:** 2024-03-27

**Authors:** Laura VanPuymbrouck, Emily M. Chun, Elizabeth D. Hesse, Kelsey Ranneklev, Camila Sanchez

**Affiliations:** ^1^Department of Occupational Therapy, Rush University, 600 S. Paulina St., Chicago, IL 60612, USA; ^2^Rush University, Chicago, USA

## Abstract

**Background:**

Developing client self-advocacy is in occupational therapy's (OT) scope of practice; however, there is limited understanding of if, or how, occupational therapists learn about self-advocacy interventions as well as implement self-advocacy into clinical practice.

**Objective:**

This study sought to identify if and how therapists learn about self-advocacy intervention approaches and identify if and how therapists implement self-advocacy into their work with clients.

**Method:**

A survey was distributed via email to academic and professional listservs in the United States, and data were collected using REDCap survey software. Descriptive statistics were analyzed data using REDCap/SPSS. Comparative statistics, Kruskal-Wallis's tests, Chi-square tests for independence, and Pearson's correlation tests analyzed differences across groups of respondents.

**Results:**

Practicing and licensed occupational therapists (*n* = 138) across the United States completed the survey. Findings indicate a majority (59.5%) of occupational therapists not learning strategies for addressing or developing client self-advocacy. Of significance, 21.7% of participants had never been exposed to concepts of client self-advocacy in academic or clinical education. Practitioners who did address self-advocacy did so indirectly through teaching-related skills (76.6%).

**Conclusion:**

Many clients of OT will need self-advocacy skills in order to address issues of exclusion and discrimination that prohibit full participation in society. Occupational therapists must prioritize incorporating client self-advocacy into curricula and clinical practice.

## 1. Introduction

A recent census report in the United States found that more than 61 million adults in the United States reported living with a disability, representing more than one-quarter of the American population [1]. Globally, upon last count, the World Health Organization (WHO) estimated the number of people with disability to be over one billion [2]. The WHO defines disability as the outcome of the interaction between individuals with a health condition and personal and environmental factors [2]. People with disabilities (PWD) experience multiple barriers that prohibit full participation in daily activities [3, 4]. For many people who identify as being disabled, self-advocacy is necessary to confront these barriers in order to access activities that they need and want to participate in [5].

The core philosophy of the OT profession is to promote and enable occupational engagement to support health and well-being, and many professional organizations have expanded their scope of practice to address social and occupational injustices that create occupational imbalance, marginalization, deprivation, and alienation [6, 7]. Additionally, practice models and frameworks increasingly describe methods to address social determinants that impact health and wellness [8] including addressing the enablement skill of advocacy with and for clients [9]. However, specifics on how to foster an individual client's ability to self-advocate are under explored.

In the newest edition of the American Occupational Therapy Practice Framework (OTPF-4), client self-advocacy is described as “Advocacy for yourself, including making one's own decisions about life, learning how to obtain information to gain understanding about issues of personal interest or importance, developing a network of support, knowing one's rights and responsibilities, reaching out to others when in need of assistance and learning about self-determination” ([6], p. 83). Despite a call to attend to client self-advocacy skills thirty years ago [10], the OTPF only added self-advocacy as an intervention area in 2014 during its third revision [11]. The occupational therapist's role in self-advocacy was described as supporting and promoting clients as they “seek and obtain resources to fully participate in daily life occupations” ([11], p. 30). These additions were made in order to reflect the most current modifications, innovation, and advancement in the profession's practices and fields [11].

Within the disability community, acquiring skills in self-advocacy are aligned with concepts of self-determination and seen as a means to challenge society's negative stereotypes and perceptions of disability [12]. Disability advocate and scholar Carol Gill asks rehabilitation professionals to consider “how would rehabilitation look if stigma resistance were recognized and cultivated as a key activity of daily living” for people with disabilities ([13], p. 999). Additionally, the social model of disability recognizes that social structures and attitudes of society disable full participation of people with disabilities that often demand the person self-advocate [14]. Because self-advocacy is often considered a facet of self-determination, the two terms can be difficult to distinguish. In contrast to self-advocacy, self-determination is characterized by the opportunity and space for an individual to have autonomy to make their own choices and control their lives [15]. Whereas self-advocacy is a process where individuals have the knowledge and skills to act to protect their rights [6].

There is a small body of evidence that occupational therapy practitioners (OTPs) recognize the importance of developing client's self-advocacy. In a qualitative study interviewing 13 occupational therapists, the researchers found that while the therapists were “willing to advocate on behalf of the clients, their preference was to use strategies with the clients, such as discussion, encouragement, problem solving, and role-playing, to allow their clients to advocate on their own” ([16], p. 349). These OTPs recognized that learning self-advocacy skills resulted in clients being empowered, not only in their current situation, but also in future scenarios long after occupational therapy services are terminated [16].

Other research also suggests that occupational therapists might have a positive impact in working with clients to develop self-advocacy skills. Shea and Jackson [17] investigated the effects of client-centeredness and occupation-based practice in six participants 15 to 18 years of age who experienced barriers to occupation. These researchers found that the implementation of interventions to develop self-advocacy skills, such as assertive communication, allowed at-risk youth to “negotiate educational formalities while coping with personal trials” and “prompted their report of more positive school experiences” [17].

An example of an OT intervention focused on developing client's self-advocacy skill development is a project called “Teens Making Environment and Activity Modifications” (TEAM) [18]. TEAM is a problem-solving self-advocacy intervention aimed at teaching young people with developmental disabilities ages 14 to 21 years to identify barriers and supports in their environment, generate modification strategies to overcome barriers, and request accommodations. The outcome of the TEAM interventions included that it supported goal achievement, school participation, and self-determination among youth with developmental conditions [18].

The most recent evidence of the link between OT interventions and self-advocacy is found in two scoping reviews of the literature. Schmidt et al. [19] sought to identify interventions within occupational therapy that could be used to improve the client's capacity to self-advocate. These researchers found that occupational therapists' unique skill sets, such as therapeutic use of self, make occupational therapists well-suited to promote the development of self-advocacy skills in their clients [19]. This review found that client-centeredness, empathy, and communication skills encompassed by one's therapeutic use of themselves allowed therapists to efficiently promote their client's understanding of skills fundamental to self-advocacy [11, 20]. Furthermore, these researchers highlighted that the occupational therapist's role in supporting a client's self-advocacy is supported by their expertise in task analysis, group dynamics, assistive technologies, and culturally competent communication [19].

The other scoping review examined the current evidence within OT and other allied health professions of the quality, characteristics, and effectiveness of interventions addressing client development of self-advocacy [21]. The findings identified that clients across different disability types benefited from interventions to support and strengthen their capacity to self-advocate. However, the majority of the articles included in the review were from other allied health professionals, not occupational therapy. These authors call on the profession to expand research on the value of addressing client self-advocacy and its role in maximizing client's occupational participation [21].

Research from outside the profession of OT suggests that self-advocacy skill development is being targeted toward several populations. These populations include students with disabilities [22] and individuals with mental illness [23]. Additionally, emerging research related to self-advocacy has been conducted with individuals with cancer [24] and individuals with acute brain injuries [25].

Emerging research documents the benefits that self-advocacy interventions can have in developing skills critical for successful self-advocacy for a variety of patient populations. Furthermore, the literature that exists supports the role of OT in designing and delivering interventions to develop the client's capacity to self-advocate. However, although self-advocacy is defined within the United States professional accreditation standards, there is no standard aligned to developing competencies in promoting client self-advocacy for students [26]. Without a standard aligned to self-advocacy, little is known about if and how occupational therapy students learn to address self-advocacy and develop competency in doing so. There is also a dearth of research that examines how OTPs acquire the knowledge to facilitate the development of self-advocacy skills in a client and how they implement the development of self-advocacy skills in client intervention. Therefore, the objectives of this study were to identify if and how occupational therapists learn about self-advocacy intervention approaches and second, to identify if, when, and how occupational therapists implement self-advocacy into treatment.

## 2. Methods

To address the research objectives, this exploratory study used a quantitative research design, utilizing an electronic survey distributed through Research Electronic Data Capture (REDCap). REDCap is a secure, web-based software platform designed to support data capture for research studies, providing valid data capture, tracking, integration, and export procedures [27]. Participants were recruited after approval of the Institutional Review Board (ORA# 20060303-IRB01), a research ethics committee of the authors' university.

### 2.1. Participants

Snowball sampling methods were used to distribute the survey by email to academic listservs and professional organizations. Academic programs received the study recruitment email and were asked to forward this to their program listservs. The inclusion criteria for this study were currently licensed occupational therapists practicing in the United States. The exclusion criteria included occupational therapy assistants and occupational therapists who do not currently practice in the United States. The email included the purpose of the study, a confidentiality statement, and that beginning the survey constituted consent. Respondents to the email who participated in the survey did so voluntarily.

### 2.2. Measures

The survey was developed based on a review of the literature on interventions focusing on the development of client self-advocacy, as well as by language on interventions found in the OTPF-4. Survey questions were broken into three sections: demographics, education and knowledge of client self-advocacy, and characteristics of interventions targeting client self-advocacy. A pilot of this survey was reviewed by three occupational therapy researchers with experience in survey development. Based on the feedback from this group, the survey was revised to address the issues of language consistency, scaling of the questions, and considerations of appropriate ethnicity options (see Table 1).

### 2.3. Data Analysis

Descriptive statistics were used to analyze the data using REDCap/SPSS allowing review of the percentages of the responses to each survey question. Comparative statistics, Kruskal-Wallis's tests, Chi-square tests for independence, and Pearson's correlation tests were also used to analyze differences among the demographics of the respondents.

## 3. Results

### 3.1. Participant Demographics

A total of 138 occupational therapists completed the survey. Participants identified primarily as females (*n* = 129), white (*n* = 126), and non-Hispanic or Latino (*n* = 127). Eleven participants chose not to disclose their gender, ethnicity, and/or race. The survey participants reported a wide variety of practice areas with the most represented being physical disability (*n* = 71), followed by pediatrics (*n* = 28), and mental health (*n* = 26). Participants reported a wide range of years of experience (from zero to more than 20), with the majority having zero to five years of OT work experience (*n* = 63). Participants also had a range of levels of education (from bachelor's degrees to PhDs), with the majority having a master's in occupational therapy (*n* = 99).

### 3.2. Descriptive Analysis

#### 3.2.1. Education on Client Self-Advocacy

When asked if they could define self-advocacy, the majority of the survey participants agreed (74.6%). When asked if training to develop skills in promoting self-advocacy had been undertaken in academic or clinical education, most participants indicated that they had been educated on the development of client self-advocacy (78.2%), but only 18.8% of participants had learned concepts and strategies/interventions to address self-advocacy with clients in practice. However, 37% of the participants reported that they were introduced to the concepts of self-advocacy but not strategies/interventions to address self-advocacy with clients, and 22.5% of the participants reported that they learned that the concepts of client self-advocacy were “somewhat” important but not taught strategies for use in practice. Of significance, 21.7% of participants reported never discussing the concept of self-advocacy or interventions for developing client self-advocacy in academic or clinical education. When asked how self-advocacy education occurred, 47.8% of the participants reported that they had learned through their own research and experience, 19.6% learned in their occupational therapy academic programs, 13% learned from their colleagues, and 7.2% learned through continuing education. However, 12.3% of the participants reported not learning any strategies to address self-advocacy.

#### 3.2.2. Perceptions on Using Self-Advocacy

**Figure 1:**
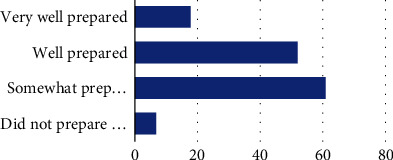
How well prepared do you feel to address self-advocacy with your clients? Note: very well prepared (18, 13.0%), well prepared (52, 37.7%), somewhat prepared (61, 44.2%), and did not prepare me at all (7, 5.1%).

**Figure 2:**
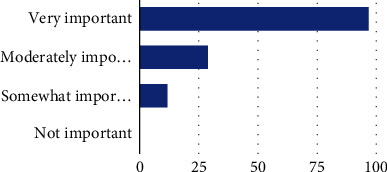
How important is it for occupational therapists to address self-advocacy in treatment sessions? Note: very important (97, 70.3%), moderately important (29, 21.0%), somewhat important (12, 8.7%), and not important (0, 0.0%).

**Figure 3:**
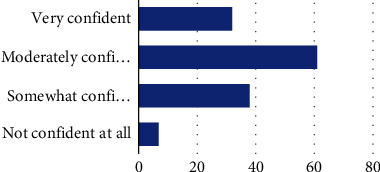
How confident do you feel in your ability to address self-advocacy during treatment? Note: very confident (32, 23.2%), moderately confident (61, 44.2%), somewhat confident (38, 27.5%), and not confident at all (7, 5.1%).

When asked how well prepared they felt to address self-advocacy with clients, the majority of participants (44.2%) reported feeling “somewhat prepared,” 13% felt “very well prepared,” 37.7% felt “well prepared,” and 5.1% felt they were “not at all prepared” (Figure 1). Regarding perceived importance of addressing self-advocacy in intervention sessions, 70.3% of participants reported this as “very important,” while 21% reported it as “moderately important” and 8.7% reported it as “somewhat important” (Figure 2). The majority of participants felt “moderately confident” (*n* = 61, 44.2%) and “somewhat confident” (27.5%) about their perceived ability to address self-advocacy during intervention, while 23.2% of participants felt “very confident” and 5.1% of participants felt “not confident at all” (Figure 3).

#### 3.2.3. Self-Advocacy Use in Interventions

**Figure 4:**
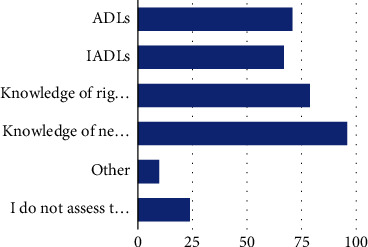
Tools to determine if self-advocacy intervention is needed. Note: ADLs (71, 51.8%), IADLs (67, 48.9%), knowledge of rights (79, 57.7%), knowledge of needs (96, 70.1%), other (10, 7.3%), and I do not assess the need for self-advocacy as a client factor (24, 17.5%).

**Figure 5:**
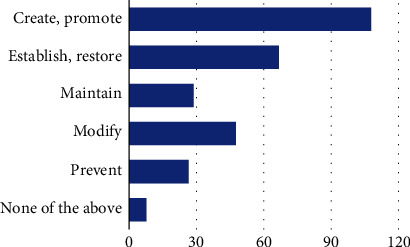
What intervention approaches do you find to be most successful in addressing self-advocacy? Note: create and promote (108, 78.8%), establish and restore (67, 48.9%), maintain (29, 21.2%), modify (48, 35.0%), prevent (27, 19.7%), and none of the above (8, 5.8%).

**Figure 6:**
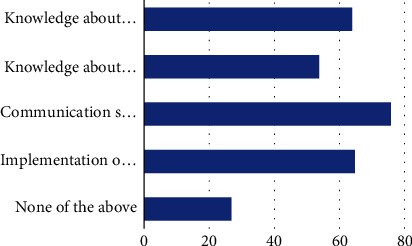
What outcome measure do you use to assess change in self-advocacy as a client factor? Note: knowledge about needs in a given ADL/IADL (64, 46.7%), knowledge about rights to accommodation (54, 39.4%), communication strategies (76, 55.5%), implementation of accommodation needs (65, 47.4%), and none of the above (27, 19.7%).

In relation to participants' responses regarding the percentage of clients for whom they evaluated the need for self-advocacy intervention, the majority of participants reported “half or more of my clients” (39.1%); 18% of the participants assessed the need for self-advocacy in all their clients, 31.2% report “less than half” of their clients, and 10.9% report that they do not assess the need for self-advocacy in any of their clients. A variety of methods were used by participants to determine whether self-advocacy of the client should be addressed in therapy (Figure 4), and the majority of participants do not specifically write self-advocacy into long- and short-term goals (75.4%). However, the majority of participants reported addressing self-advocacy indirectly through teaching-related skills (76.6%) such as communicating needs, followed by education of rights (51.1%). If participants identified addressing self-advocacy in interventions, they used a diverse set of approaches (Figure 5) as well as outcomes to assess change in client self-advocacy (Figure 6).

### 3.3. Comparative Analysis

This study used SPSS, Kruskal-Wallis' tests, Chi-square tests for independence, and Pearson Correlation tests to compare the respondents' answers to questions. Non-parametric tests were required due to the relatively small sample size and unequal distribution of the sample. Self-reported preparedness, perceived importance, self-reported confidence, and self-reported frequency of use are based on responses to questions 12, 13, 14, and 15, respectively (Table 1). A Kruskal-Wallis test revealed that when participants were grouped according to the amount of education received on self-advocacy interventions, there was a significant difference in the frequency of use of self-advocacy interventions (*p* = 0.036) and a significant difference in feelings of readiness (*p* = 0.010). However, there is no significant difference in confidence or perceived importance when grouped by the reported amount of education received on self-advocacy interventions.

Participants were also grouped by degree level—bachelor's, master's, clinical doctorate, and PhD—to determine if there is a difference in feelings of preparedness, confidence, frequency of use, and perceived importance between groups. A Kruskal-Wallis test indicated a significant difference when participants were grouped by degree level in self-reported feelings of preparedness (*p* = 0.035) and confidence (*p* = 0.029) with a higher degree level associated with increases in both; however, there is no significant difference in self-reported frequency of use of self-advocacy interventions or in perceived importance when grouped by degree level.

When grouped by response to Q8 “Can you define self-advocacy as a client issue in occupational therapy?” (Table 1), a Kruskal-Wallis test indicated a significant difference in self-reported preparedness (*p* ≤ 0.001), perceived importance (*p* ≤ 0.001), self-reported confidence (*p* ≤ 0.001), and self-reported frequency of use (*p* ≤ 0.001) (see Table 2). There was also a significant positive correlation between the ability to define self-advocacy and frequency of use (*p* ≤ 0.001), confidence (*p* ≤ 0.001), preparedness (*p* ≤ 0.001), and perceived importance (*p* ≤ 0.001) (see Table 3). Participants who reported a greater ability to define client self-advocacy were more likely to self-report increased frequency of use, preparedness, importance, and confidence. When participants were grouped by years of practice into five or less years of practice and six or more years of practice, a Chi-square test indicated that there was a significant difference in the knowledge of self-advocacy concepts and strategies in their education (*X*^2^_0.05,1_ = 7.178381, *p* = 0.007). Participants with up to five years of experience were more likely to report that they had learned concepts and strategies for developing client self-advocacy in their education. When participants were grouped by areas of practice, a Kruskal-Wallis test indicated that they did not have significant differences in the area of practice and in having learned concepts and strategies in their education.

## 4. Discussion

Many occupational therapy clients have lifelong disabilities that result in barriers to full participation in society requiring the knowledge and skills to advocate for themselves [5]. The objectives of this exploratory study were to (1) identify if, and how, occupational therapists learn about self-advocacy intervention approaches and (2) to identify if, when, and how occupational therapists implement client self-advocacy interventions in their intervention plans. The findings of this study build upon preliminary reports of the findings of this research presented at an OT national conference [28] and clarify how occupational therapists are exposed to and learn to address and foster development of client self-advocacy. Participants' responses indicated that most had been introduced to or “somewhat” introduced to concepts of self-advocacy but not strategies for intervention design. The combination of these responses (59.5%) indicates that a majority of occupational therapists had not learned strategies for addressing or developing client self-advocacy. In fact, one-fifth of the participants had never been exposed to concepts of client self-advocacy in their academic or clinical education. Similar findings can be found in the literature on school counselor experiences in exposure and knowledge of the principles of self-advocacy and self-advocacy counseling of minority students and students with disabilities [29]. Working with students to learn strategies of self-advocacy to address social injustices is supported by school counselors; however, this research suggested that academic programing did not prepare counselors with the knowledge on how to do so [29].

Despite being recognized by the OTPF-4 as important, the majority of this study's participants were not exposed academically to methods or intervention strategies to develop client self-advocacy skills. Professional intervention courses may continue to focus more on biomechanical or rehabilitative approaches to client care versus social barriers to participating in occupations. This is supported by recent research that finds greater support for the medical versus social model of disability as a locus of intervention by occupational therapists, both in theory and in practice [30].

The limited academic exposure to client self-advocacy found in this study could result from the fact that self-advocacy has only been part of the OTPF since 2014 (third edition), and approximately half of the participants reported more than 5 years of practice. What is encouraging is almost half (47.8%) of the participants researched their own approaches to address the development of client self-advocacy skills. This finding is supported by research of occupational therapists during their first year of practice feeling well prepared to seek out information that they needed to support ongoing or lifelong learning [31].

The second objective of this study was to examine if, when, and how occupational therapists were addressing client self-advocacy. Of interest is that the majority of participants (70.3%) reported that addressing self-advocacy with clients is “very important”; however, the majority do not specifically write self-advocacy in client treatment goals (75.4%). This finding suggests that occupational therapists may be addressing client self-advocacy as an underground practice [32] a result which is similar to a study exploring if practitioners are implementing client empowerment into practice [33]. In that study [33], participants recognized the need to implement empowerment-focused interventions but used documentation language focused on traditional areas of intervention such as increased participation in instrumental activities of daily living (I/ADL) without directly describing empowerment as a strategy. Similarly, the participants in this study described the way in which they addressed self-advocacy indirectly through teaching-related skills (76.6%), such as communication or participation in I/ADLs.

In response to the question of how self-advocacy was addressed with clients, more than half of the participants described educating clients on their civil rights. This is relevant because previous research describes understanding of disability rights and learning to take personal action, including communicating accommodation needs, and is critical to a person with disability's agency in self-advocacy [34]. Furthermore, communication strategies and the implementation of accommodations were outcomes most often described by participants as assessments of change in the self-advocacy of clients. This is encouraging and potentially points to growing attention by practitioners to include interventions that focus on physical and socially constructed barriers that disable clients from participating in meaningful occupations, compared to research finding otherwise. In a study on knowledge of the Americans with Disability Act (ADA) and education of clients on acting upon their ADA rights, Redick et al. [35] found that the majority of OTPs had little knowledge on the ADA and are not acting to implement Title III provisions. These researchers [35] claimed that without knowledge of the ADA OTPs are unable to work with clients to self-advocate based on their ADA rights, and this directly impacted the goal of living independently due to the continued presence of barriers to participation. The ADA is only one of many disability laws or acts that exist in many countries around the world that work to eliminate discrimination and barriers against people with disabilities [36]. It is difficult to determine whether knowledge and rights of the disability laws and acts of a country have become more of a consideration across occupational therapy practice in the twenty plus years since Redick et al.'s [35] research; however, the findings of this current study suggest that they may be. Future research on this issue would be beneficial.

When comparing differences between groups of respondents, the findings show the amount of exposure to self-advocacy interventions and the ability to define self-advocacy both significantly related to the use of self-advocacy interventions. Additionally, when looking at the level of educational degree, those OTPs with higher levels of degree perceived feeling more prepared and confident in attending to self-advocacy; however, this did not translate into implementing self-advocacy into interventions. Other research identifies that clinical mentorship plays a role in advancing practitioner clinical reasoning that may explain these differences [37, 38]. It may be that clinical mentors who focus on client self-advocacy provide examples for practitioners to do so in their own intervention planning. Adult learning principles support that despite having a strong knowledge of concepts through academic preparation, translating these into practice requires active engagement from the learner [39]. It is possible that practitioners who learn by observing others or who independently seek out information on client self-advocacy have a greater level of knowledge of the value of how self-advocacy can impact a client's future capacity for full participation in everyday life and therefore are more likely to implement it into practice.

This study found a significant difference between practitioner responses to being exposed to self-advocacy when grouped by years of practice. There was a significant difference in participants with 0-5 years of experience and 6 or more years in their frequency in answering “yes” to learning self-advocacy concepts and strategies while in university. It may be that exposure to self-advocacy in academia and clinical experiences has increased specifically due to its inclusion in the OTPF in 2014. If academic programs increasingly expose students to strategies for developing client self-advocacy, this may stimulate greater use of self-advocacy interventions in the future.

### 4.1. Implications for OT Practice

The distinct value of occupational therapy as part of any health care team is the role it plays in supporting clients' capacity to achieve full participation in society. However, although the social model of disability has been introduced within the profession, concepts of exclusion, discrimination, and microinequities experienced every day by PWD that exclude full participation may not be well understood by OT educators or practitioners. Occupational therapy researchers [40] have identified a dearth of research on OTPs' understanding of approaches to empowerment, advocacy, and the sociopolitical barriers described in the academic area of disability studies and by disability advocates. Many clients of OT focus on goals addressing basic activities of daily living (ADL) seen before as skills seen as essential prerequisites to engaging and participating with others in society. However, very often, PWD must also have the capacity to perform the essential skills of self-advocacy to achieve full participation. As a profession, we recognized this critical factor and have included it into some of our professional frameworks. However, integration of self-advocacy in interventions is still in its infancy. The findings from this study expose the need to weave self-advocacy into education, research, and practice. Recommendations include the following:

- Providing opportunities for OTPs to gain an understanding of the sociopolitical context of the lives and worlds our clients live in, through attending disability culture or activism events, or joining disability-based advocacy groups. By doing so, we will have a more holistic understanding of the critical value of supporting the client's capacity to advocate [41].
- Incorporating experts in living with disability into course design and implementation
- Infusing interventions to develop client self-advocacy and discourse from disability advocacy groups, disability studies, disability rights, disability justice, and disability culture into university program designs
- Develop and conduct occupational therapy-specific research investigating the effectiveness of client self-advocacy interventions
- Include into professional practice standard requirements to obtain knowledge that would support developing client self-advocacy. A place to start would be requiring a working knowledge on national civil and legal rights of PWD as part of the students' educational outcomes

### 4.2. Limitations

This study has several limitations, including a possible sampling bias due to the sample size and limited geographical representation. Given that academic listservs were used as a means of recruitment, there may be a socioeconomic bias of the participants. Survey questions were developed by the research team, and while attempts were made to ensure that questions were clear and understandable, participants may have misinterpreted the meaning of a question. This study was also an exploratory study, and therefore, interpretation of its findings should occur with caution. Finally, the diversity of participants was limited, with the majority of participants being white and female; however, this is representative of the demographic of the profession [42].

## 5. Conclusion

The findings of this study suggest that occupational therapists have limited awareness of the need to address self-advocacy intervention planning, as well as limited strategies to foster the development of client self-advocacy. However, many OT clients will need self-advocacy skills in order to target issues of exclusion and discrimination that prohibit full participation in society. Importantly, many participants reported limited exposure to interventions to support client self-advocacy in their academic programs. Self-advocacy as a consideration when working with clients was added to our scope of practice only recently. To fully embrace self-advocacy, occupational therapy educators and practitioners must first understand the many and persistent barriers that continue to prohibit full inclusion of people with disabilities in society. In recognizing this, efforts should focus on prioritizing client self-advocacy as a necessary and vital activity of daily living and work toward developing evidence-based and effective interventions to support clients' capacity to self-advocate.

## Figures and Tables

**Table 1:** Study questionnaire items.

Question	
Q1. Gender	(A) Male (B) Female (C) Gender nonconforming (D) Prefer not to say

Q2. Race (select all that apply)	(A) American Indian or Alaska Native (B) Asian (C) Black or African American (D) Native Hawaiian or Other Pacific Islander (E) White (F) Prefer not to say

Q3. Ethnicity	(A) Hispanic or Latino (B) Not Hispanic or Latino (C) Prefer not to say

Q4. Area of practice (select all that apply)	(A) Physical disability (B) Mental health (C) Community practice (D) School based (E) Pediatrics (F) Other

Q5. What is your highest degree level?	(A) Bachelor's in occupational therapy (B) Master's in occupational therapy (C) Doctorate in occupational therapy (D) PhD

Q6. What state do you primarily practice in? (drop-down menu)	

Q7. How long have you been practicing as an OT?	(A) 0-5 years (B) 6-10 years (C) 10-15 years (D) 16-20 years (E) 20+ years

Q8. Can you define self-advocacy as a client issue in occupational therapy?	(A) Yes, and I can explain it well to other people (B) Yes, but I cannot explain it well to others (C) Somewhat, I have heard of the concept but I am unsure of how to define it (D) Not well

Q9. Can you differentiate between self-determination and self-advocacy?	(A) Yes, I understand the difference and can explain it to other people (B) Yes, I know these are different concepts but find it difficult to explain to others (C) Somewhat, I have heard these described as being different but unsure of how (D) Not well

Q10. In your academic or clinical education, were interventions on developing self-advocacy in clients introduced?	(A) Yes, I learned concepts as well as strategies/interventions for addressing self-advocacy as a client factor for use in practice (B) Yes, I was introduced to concepts of self-advocacy as a client factor but not strategies for use in practice (C) Somewhat, concepts of self-advocacy as being important were discussed but not as a client factor to intervene upon (D) No, self-advocacy as a client factor was never presented to me in my academic or clinical education

Q11. If you learned strategies for addressing self-advocacy, how did this occur?	(A) Occupational therapy school (B) Continued education (C) Colleagues (D) Through my own research/experience (E) I have not learned strategies

Q12. How well prepared do you feel to address self-advocacy with your clients?	(A) Very well prepared (B) Well prepared (C) Somewhat prepared (D) Did not prepare me at all

Q13. How important is it for occupational therapists to address self-advocacy in treatment sessions?	(A) Very important (B) Moderately important (C) Somewhat important (D) Not important

Q14. How confident do you feel in your ability to address self-advocacy during treatment?	(A) Very confident (B) Moderately confident (C) Somewhat confident (D) Not confident at all

Q15. In how many clients do you assess the need for self-advocacy?	(A) All of my clients (B) Half or more of my clients (C) Less than half of my clients (D) None of my clients

Q16. What tools do you use to determine if self-advocacy is a client factor in need of intervention? (select all that apply)	(A) ADLs (B) IADLs (C) Knowledge of rights (D) Knowledge of needs (E) Other: ___________ (F) I do not assess the need for self-advocacy as a client factor

Q17. Is self-advocacy specifically written into your long- and short-term goals?	(A) Yes, I include self-advocacy in long- and short-term goals (B) No, I do not include self-advocacy in long- or short-term goals

Q18. If you address self-advocacy, which area of occupation is this most often linked to? (select all that apply)	(A) ADLs (B) IADLs (C) Rest and sleep (D) Education (E) Work (F) Play (G) Leisure (H) Social participation (I) I do not address self-advocacy with clients

Q19. In what ways do you address self-advocacy? (select all that apply)	(A) Directly as a part of therapy sessions (B) Indirectly through teaching skills that relate to self-advocacy (C) Education of rights (D) Other: _____________ (E) I do not address self-advocacy

Q20. What intervention approaches do you find to be most successful in addressing self-advocacy? (select all that apply)	(A) Create, promote (B) Establish, restore (C) Maintain (D) Modify (E) Prevent (F) None of the above

Q21. What outcome measure do you use to assess change in self-advocacy as a client factor? (select all that apply)	(A) Knowledge about needs in a given ADL/IADL (B) Knowledge about rights to accommodation (C) Communication strategies (D) Implementation of accommodation needs (E) None of the above

**Table 2:** Ability to define self-advocacy and frequency of use, confidence, preparedness, and importance.

	Frequency of use	Feeling prepared	Confidence	Importance
Kruskal-Wallis's *H*	21.733	36.501	40.835	19.013
df	3	3	3	3
Asymp. sig.	0.000^∗^	0.000^∗^	0.000^∗^	0.000^∗^

Note. A Kruskal-Wallis test. Grouping variable is the ability to define self-advocacy. ^∗^*p* ≤ 0.001.

**Table 3:** Correlations between frequency of use, ability to define, feeling of preparedness, perceived importance, and confidence.

		Frequency of use	Ability to define	Feeling prepared	Perceived importance	Confidence
Frequency of use (Q15)	Pearson's correlation	1	0.392^∗∗^	0.542^∗∗^	1. 0.412^∗∗^	0.542^∗∗^
	Sig. (2-tailed)		0.000	0.000	.0000	.0000
	*N*	138	138	138	138	138

Ability to define (Q8)	Pearson's correlation	0.392^∗∗^	1	0.478^∗∗^	0.349^∗∗^	0.551^∗∗^
	Sig. (2-tailed)	0.000		0.000	0.000	0.000
	*N*	138	138	138	138	138

Feeling prepared (Q12)	Pearson's correlation	0.542^∗∗^	0.478^∗∗^	1	0.438^∗∗^	0.805^∗∗^
	Sig. (2-tailed)	0.000	0.000		0.000	0.000
	*N*	138	138	138	138	138

Perceived importance (Q13)	Pearson's correlation	0.412^∗∗^	0.349^∗∗^	0.438^∗∗^	1	0.508^∗∗^
	Sig. (2-tailed)	0.000	0.000	0.000		0.000
	*N*	138	138	138	138	138

Confidence (Q14)	Pearson's correlation	0.542^∗∗^	0.551^∗∗^	0.805^∗∗^	0.508^∗∗^	1
	Sig. (2-tailed)	0.000	0.000	0.000	0.000	
	*N*	138	138	138	138	138

Note: Pearson's correlation. Questions found in Table 1. ^∗∗^Correlation is significant at the 0.01 level (2-tailed).

## Data Availability

All data is provided in full in the results section of this paper.
